# Environmental niche overlap in sibling planktonic species *Calanus finmarchicus* and *C. glacialis* in Arctic fjords

**DOI:** 10.1002/ece3.9569

**Published:** 2022-12-08

**Authors:** Agata Weydmann‐Zwolicka, Finlo Cottier, Jørgen Berge, Sanna Majaneva, Piotr Kukliński, Adrian Zwolicki

**Affiliations:** ^1^ Department of Marine Plankton Research, Institute of Oceanography University of Gdansk Gdynia Poland; ^2^ Department of Arctic and Marine Biology, Faculty of Biosciences, Fisheries and Economics The Arctic University of Norway Tromsø Norway; ^3^ Scottish Association for Marine Science Oban UK; ^4^ Department of Biology, Faculty of Natural Sciences Norwegian University of Science and Technology Trondheim Norway; ^5^ Department of Ecology Institute of Oceanology, Polish Academy of Sciences Sopot Poland; ^6^ Department of Vertebrate Ecology and Zoology, Faculty of Biology University of Gdansk Gdańsk Poland

**Keywords:** Atlantification, ecological niche, plasticity, Kongsfjorden, Rijpfjorden, Svalbard, zooplankton

## Abstract

Knowledge of environmental preferences of the key planktonic species, such as *Calanus* copepods in the Arctic, is crucial to understand ecosystem function and its future under climate change. Here, we assessed the environmental conditions influencing the development stages of Atlantic *Calanus finmarchicus* and Arctic *Calanus glacialis*, and we quantified the extent to which their niches overlap by incorporating multiple environmental data. We based our analysis on a 3‐year seasonal collection of zooplankton by sediment traps, located on moorings in two contrasting Svalbard fjords: the Arctic Rijpfjorden and the Atlantic‐influenced Kongsfjorden. Despite large differences in water temperature between the fjords, local realized ecological niches of the sibling *Calanus* species overlapped almost perfectly. The exception was the earliest copepodites of *C. glacialis* in Rijpfjorden, which probably utilized the local ice algal bloom in spring. However, during periods with no sea ice, like in Kongsfjorden, the siblings of both *Calanus* species showed high synchronization in the population structure. Interestingly, differences in temperature preferences of *C. finmarchicus* and *C. glacialis* were much higher between the studied fjords than between the species. Our analysis confirmed the high plasticity of *Calanus* copepods and their abilities to adapt to highly variable environmental settings, not only on an interannual basis but also in a climate warming context, indicating some resilience in the *Calanus* community.

## Graphical abstract

We assessed environmental conditions influencing the development stages of Atlantic *Calanus finmarchicus* and Arctic *Calanus glacialis*, and we quantified the extent to which their niches overlap based on a three‐year seasonal collection of zooplankton by sediment traps, located on moorings in two contrasting Svalbard fjords: the Arctic Rijpfjorden and the Atlantic‐influenced Kongsfjorden. Despite large differences in water temperature between the fjords, local realized ecological niches of the sibling *Calanus* species overlapped almost perfectly. Our analysis confirmed the high plasticity of *Calanus* copepods and their abilities to adapt to highly variable environmental settings, not only on an interannual basis but also in a climate warming context, indicating some resilience in the *Calanus* community.

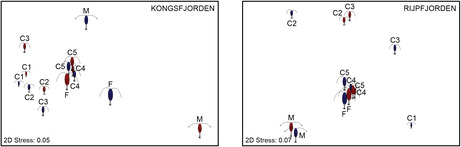

## 1 INTRODUCTION

Planktonic copepods of the genus *Calanus* are important for the functioning of both marine and terrestrial Arctic ecosystems. This is due to their role in the lipid‐based energy flux in the Arctic, converting low‐energy carbohydrates and proteins in ice algae and phytoplankton into high‐energy wax esters available for higher trophic levels (Falk‐Petersen et al., 2009). The congener species *Calanus finmarchicus* and *Calanus glacialis* are among the main contributors to the zooplankton biomass in Arctic shelf seas (Aarflot et al., 2018). Due to their high lipid content, they serve as the main food source for numerous consumers such as fish (Varpe et al., 2005), seabirds (Karnovsky et al., 2003; Kwasniewski et al., 2012; Stempniewicz et al., 2021), and baleen whales (Berge et al., 2012).

*Calanus glacialis* is an Arctic endemic species with a panmictic population with large‐scale gene flow across the peripheral seas of the Arctic Ocean (Weydmann et al., 2016). The boreal *C. finmarchicus* has its center of distribution in the large gyres of the North Atlantic, but it is regularly transported with ocean currents of Atlantic origin to the Arctic, where it can reach high biomass (Aarflot et al., 2018; Carstensen et al., 2012; Kosobokova & Hirche, 2009). The life cycle of both species includes an overwintering phase at depth, while their reproduction, growth, and development are closely coupled to the short but intense algal blooms in surface waters during spring and summer. During the summer, they build up their large lipid reserves, which sustain them through the winter and fuel molting and maturation in spring. Differences in life history strategies between the two species reflect adaptations to the environmental settings in their core areas of distribution (Falk‐Petersen et al., 2009).

*Calanus glacialis* is adapted to the environmental conditions of seasonal ice‐covered seas, where low temperatures prevail and the onset and duration of the phytoplankton spring bloom can vary substantially between years, but which may also provide an additional food source early in spring in the form of an ice algal bloom (Daase et al., 2013; Søreide et al., 2010). Reproduction can be fueled on lipid reserves alone, thus enabling *C. glacialis* to initiate egg production prior to the onset of the spring bloom, but it will also utilize the ice algal bloom to increase the reproductive output. Under favorable conditions, reproduction is timed in a way that allows young developmental stages to fully utilize the phytoplankton spring bloom (Søreide et al., 2010). As the season of high food availability can be short and growth is slow at low temperatures (Weydmann et al., 2015), *C. glacialis* needs 1–2 years to fulfill its life cycle (Daase et al., 2013). The option to extend its life cycle over 2 years gives this Arctic copepod the flexibility to cope with the high environmental variability in seasonally ice‐covered seas, and allows it to grow larger and accumulate larger lipid reserves than the smaller, less lipid‐rich Atlantic *C. finmarchicus*.

*Calanus finmarchicus* is adapted to a boreal environment, where the timing of the spring bloom is more predictable, the primary production season is longer and where warmer temperatures ensure faster development, making it possible to complete the life cycle in 1 year (at the northern extend of its distributional ranges) or even less (multigenerational life cycle in the southern end of its distribution; Weydmann et al., 2018). This comes at the cost of smaller body size and smaller lipid reserves compared to the Arctic *Calanus* species, and *C. finmarchicus* therefore has to rely on external food supply to fuel reproduction in spring as its lipid reserves are not sufficient. While *C. finmarchicus* is regularly transported into the Arctic, the short pelagic algae growing season and low temperatures limit its ability to reproduce and to reach older developmental stages with sufficient lipid reserves that would allow successful overwintering in the Arctic (Hirche & Kosobokova, 2007; Ji et al., 2012). However, with climate warming leading to less sea ice, increased water temperatures, and longer algae growth season, there are indications that conditions may become more favorable for *C. finmarchicus* in the Arctic. A poleward shift and an increase of *C. finmarchicus* contribution to the overall *Calanus* biomass has been already observed in some Arctic regions (Carstensen et al., 2012; Chust et al., 2014; Hop, Assmy, et al., 2019; Møller & Nielsen, 2020; Weydmann et al., 2014), and *C. finmarchicus* may be able to accelerate its development leading to potentially produce a second generation in warmer years (Weydmann et al., 2018).

Despite these differences in life history strategies, spatial distribution of *C. finmarchicus* and *C. glacialis* largely overlaps in the European Arctic, especially in the areas that are regularly affected by the influx of Atlantic water masses (Aarflot et al., 2018; Carstensen et al., 2012; Walkusz et al., 2009; Weydmann et al., 2013; Willis et al., 2006). However, due to harsh winter conditions and sea ice coverage, studies on the sibling *Calanus* species and other important zooplankton taxa in the European Arctic are usually limited to late spring–early autumn that bias the understanding of their biology and environmental preferences. Further, studies are often based on different sampling approaches than traditional net collection (Weydmann‐Zwolicka, Balazy, et al., 2021), such as the use of sediment traps that allow for the year‐round continuous zooplankton collection (Weydmann‐Zwolicka, Prątnicka, et al., 2021; Willis et al., 2008). Both species are known to exhibit high plasticity in relation to environmental conditions (Trudnowska et al., 2020), although routinely measured hydrographic properties of water masses, such as temperature and salinity, seem to be the key in predicting their distribution in the European Arctic: *C. finmarchicus* is generally found in warmer and more saline waters of Atlantic origin, whereas *C. glacialis* is associated with colder, Arctic waters (Aarflot et al., 2018; Daase et al., 2007; Hop et al., 2002; Weydmann et al., 2013; Weydmann & Kwaśniewski, 2008). However, in some Svalbard fjords, main difference between *C. glacialis* and *C. finmarchicus* life traits was based on different timing in reproduction, what reduced species competition, although they were reported to occupy similar environmental niches (Hatlebakk et al., 2022).

Environmental niche corresponds to abiotic factors which, according to the classical definition of an ecological niche describing it as a multidimensional hypervolume in which a species maintains a viable population, influence birth and death rates together with biotic factors (Hutchinson, 1957). Species niches overlap when co‐occurring species share parts of their niche spaces with each other and, consequently, compete for resources. The degree to which species niches overlap may give insight into their interactions, e.g., high niche overlap indicates competition for resources, and may lead to exclusion for some species; while low niche overlap generally implies utilization of different resources, and thus lower levels of species interactions (Tsafack et al., 2021). Such interactions are especially important for species inhabiting regions that are exposed to rapid environmental changes, such as the recent Arctic.

It is impossible to measure all variables responsible for shared environmental niches of marine zooplankton species, thus any statistical attempts must be based on limited, and possibly routinely measured, environmental data (Beaugrand & Helaouët, 2008; Freer et al., 2022; Hatlebakk et al., 2022). The major objective of our study was to assess *C. finmarchicus* and *C. glacialis* environmental preferences, and the degree to which environmental niches of their development stages overlap, with the use of data on temperature, salinity, and chlorophyll *a* fluorescence, obtained from moorings located in two contrasting Svalbard fjords: the high‐Arctic Rijpfjorden and Atlantic‐influenced Kongsfjorden, and coupled with the 3‐year seasonal collection of zooplankton by the attached sediment traps. Based on the results, we also tried to predict the future of *Calanus* complex in a climate warming context.

## 2 MATERIALS AND METHODS

### 2.1 Study area

**FIGURE 1:**
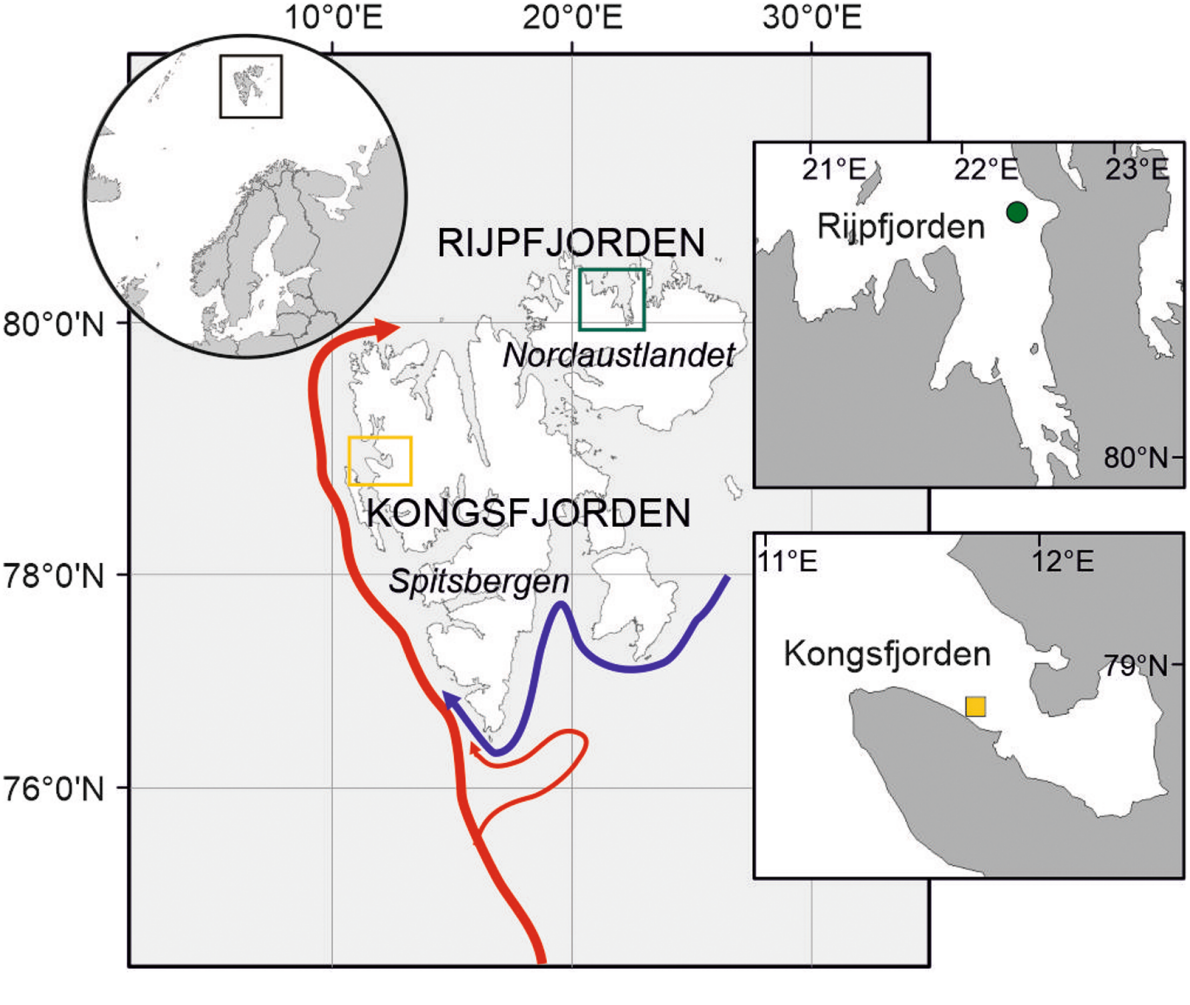
Svalbard archipelago with the main ocean currents (red, relatively warm of the Atlantic origin; blue, cold of the Arctic origin), and the positions of moorings in Rijpfjorden (green circle) and Kongsfjorden (yellow square).

Two contrasting fjords in Svalbard, a high‐Arctic archipelago, were selected as the study areas: Kongsfjorden and Rijpfjorden (Figure 1). Kongsfjorden, a west‐facing fjord of Spitsbergen, is regularly affected by influx of Atlantic water masses advected from the West Spitsbergen Current over the adjacent shelf area (Cottier et al., 2005; Promińska et al., 2017). A long‐term time series of temperature measurements in the fjord indicate that the water temperature has increased in the order of 1°C per decade since 2002 (Cottier et al., 2019). Consequently, Kongsfjorden has been mostly ice‐free since winter 2005–2006 (Cottier et al., 2007; Walczowski et al., 2012). The hydrographic settings in Kongsfjorden strongly influence the zooplankton community structure, which shows high seasonal, spatial, and interannual variation, with high abundances of Atlantic species (Hop et al., 2002; Hop, Wold, et al., 2019; Walkusz et al., 2009; Weydmann‐Zwolicka, Prątnicka, et al., 2021; Willis et al., 2006, 2008).

Rijpfjorden is a north‐facing fjord located on Nordaustlandet (80°N), it is predominantly ice‐covered for at least 6–8 months of the year and experiences oceanographic conditions that are more Arctic in character (relatively cold and fresh). Its zooplankton community is dominated by Arctic species, although species of boreal and Atlantic origin are also present, especially when waters of Atlantic origin are advected into the fjord (Hop, Assmy, et al., 2019; Weydmann et al., 2013; Weydmann‐Zwolicka, Prątnicka, et al., 2021). No significant increase in temperature has been observed in Rijpfjorden since long‐term monitoring began in 2006 (Cottier et al., 2019).

### 2.2 Sampling and laboratory work

Sediment traps (McLane Parflux 78H–21 with a 21 bottle carousel and a 0.5 m^2^ aperture) were deployed at 60 m depth on single‐point moorings in Kongsfjorden (78°57.8′N 11°47.88′E, bottom depth of 230 m) and in Rijpfjorden (80°18.1′N 22°17.4′E, bottom depth of 236 m) for three consecutive seasonal cycles from October 2014 to August 2017. The length of the sediment trap exposure times differed between seasons, from 1 week in spring and summer to 1 month in autumn and winter (details are presented in Weydmann‐Zwolicka, Prątnicka, et al., 2021). The trap sample bottles were prefilled with filtered seawater adjusted with NaCl to 35 PSU to provide a density discontinuity relative to ambient seawater and to avoid diffusion of samples. To preserve deposited material during and after deployment, 4% formalin buffered with sodium borate was added to each sample cup. Temperature (T) and salinity (S) were recorded during the sediment trap deployment period with Seabird 37 Microcats moored at a depth of 60 m. Additional Seapoint Sensors were mounted at 25 m and used to measure chlorophyll *a* fluorescence (Chl‐a), and the presence or absence of sea ice was interfered from data collected by upwards looking Acoustic Doppler Current Profilers (ADCP; Hyatt et al., 2008).

Detailed laboratory procedures of handling the sediment trap samples are described in Weydmann‐Zwolicka, Prątnicka, et al. (2021). *Calanus* individuals were identified to species based on their morphology (Brodskii et al., 1983), and prosome lengths (distance from the apex of head to the distal margin of the last thoracic segment, laterally) of individual copepodite stages (C1–C5, and adult females and males) according to Weydmann and Kwaśniewski (2008) under a stereomicroscope equipped with a calibrated ocular micrometer (Leica M125 C; Leica Imaging Systems GmbH). Due to relatively small differences in the prosome length of earlier copepodites, C1–C3 were measured at the magnification of at least × 25. Only individuals that showed no signs of decomposition, suggesting they were killed by the applied formaldehyde, were chosen for further analyses (Matsuno et al.,  2014).

### 2.3 Statistical analyses

Due to possible disturbances connected with sediment trap deployment, the first samples from each cycle (24.08–01.10) were excluded from further analyses, resulting in 20 samples collected during each year in each fjord, and a total of 120 samples. After excluding the samples in which neither *C. finmarchicus* nor *C. glacialis* was present, we obtained 100 samples, which were further analyzed. Sediment trap exposure time varied between the seasons (Weydmann‐Zwolicka, Prątnicka, et al., 2021); hence, we standardized *Calanus* abundance data to a daily flux, expressed as individual m^−2^ day^−1^, according to Matsuno et al. (2014), and these data were used in the following analyses. Because the bloom intensity differed between the years and fjords, the raw Chl‐a fluorescence values were recalculated in the range of 0–100 for each year/fjord. This way the differences in Chl‐a fluorescence values between the fjords and years did not mask the bloom timing and duration and, thus its influence on *Calanus* species phenology. For the purpose of statistical analyses, temperature, salinity, and fluorescence data were averaged for the respective periods of sediment traps exposure time. Sea ice cover was not included in the statistical analysis because it was present in Rijpfjorden only.

The relationship between environmental factors such as location (Fjord), sampling years, understood as cycles from October to August in the following year (Year), and calendar months (Month), as well as the daily flux of *C. finmarchicus* and *C. glacialis* development stages, was assessed using PERMANOVA. The model design included two fixed factors: Fjord and Month, and the interaction between them: Fjord × Month; as well as a random factor: Year (Month), and interaction between this factor and Fjord: Fjord × Year (Month). PERMANOVA was run under the following conditions: type III (partial) sums of squares, permutation of residuals under a reduced model, 999 permutations, and fixed effects sum to zero for mixed terms.

The influence of the continuous environmental variables, averaged water temperature, salinity, day length, and Chl‐a fluorescence on the studied *Calanus* copepodites daily flux, was tested with a distance‐based linear model (DistLM) routine, illustrated by distance‐based redundancy analysis (dbRDA) diagrams. The *Calanus* daily flux data were transformed [*n*′ = log (*n* + 1)] prior to further analyses, and similarities between samples were examined using the Bray–Curtis index. To build the models, all specified predictor variables were included using a forward selection procedure, and the selection criterion was based on adjusted *R* ^2^ values. Because predictor variables jointly affect species composition in marine environments, we showed only the results of sequential tests (Legendre & Anderson, 1999). Both types of analyses were performed using PRIMER 7 software package (Clarke & Warwick, 2001), with the PERMANOVA+ add on (Anderson et al., 2008). This approach allowed to provide quantitative measures of variation in *Calanus* daily flux explained by both types of predictor variables: factors and continuous variables, separately, so the analysis was not biased by possible correlations between some variables, like temperature and months.

Niche‐based models that are calibrated in the native range by relating species observations to climatic variables are commonly used to predict their future spatial extent (Broennimann et al., 2007). Statistical procedures, which were used to quantify and compare ecological niches of the North Atlantic *Calanus* copepods, have been proposed for *C. finmarchicus* and *C. helgolandicus* and were based on three environmental factors: temperature, salinity, and bathymetry (Beaugrand & Helaouët, 2008). Since the degree of *C. finmarchicus* and *C. glacialis* ecological niche overlap in the high‐Arctic fjords, in which these sibling species co‐occur, has never been quantified, we decided to calculate the average niche overlap (NO) values between the pairs of studied species and their development stages, also by incorporating three routinely measured environmental predictors: averaged water temperature and salinity, as well as standardized Chl‐a fluorescence data, which were selected based on the results of DistLM, and were analyzed separately for each fjord, Rijpfjorden and Kongsfjorden.

To calculate environmental niche overlap for *C. finmarchicus* and *C. glacialis*, we used the unified analysis of NO described in Geange et al. (2011) and the R codes provided in this publication. For each suite of analyses, we used appropriate transformations and probability models (as described in Geange et al., 2011) to measure niche overlap between the pairs of development stages and over each different niche axis, here understood as temperature, salinity, and Chl‐a fluorescence. Measurements derived from different environmental data were then combined into a single, unified analysis of NO by averaging over multiple axes, and null model permutation tests were used to assess statistical differences in niche overlap. This procedure led to directly comparable measures of NO, with the overlap statistic between two sibling *Calanus* copepods defined as the overlapping area between the distributions for each species; where NO ranged between 0 (two distributions are completely separated) and 1 (distributions exactly coincide). The obtained matrix of calculated NO dissimilarities (1‐NO) of *Calanus* development stages was then used to graphically display the results for each fjord with the use of nonmetric multidimensional scaling (nMDS), and the Spearman rank correlation between the matching resemblance matrices was tested by RELATE routine in PRIMER 7.

Afterwards, two‐way ANOVA was performed in R (RStudio Team, 2015; R CoreTeam, 2017) to test if the niche position across temperature axis, which was the most influential environmental variable according to DistLM and the most differentiating single niche axis, significantly differed between *C. finmarchicus* and *C. glacialis* and between Rijpfjorden and Kongsfjorden.

## 3 RESULTS

### 3.1 Environmental conditions in the fjords

**FIGURE 2:**
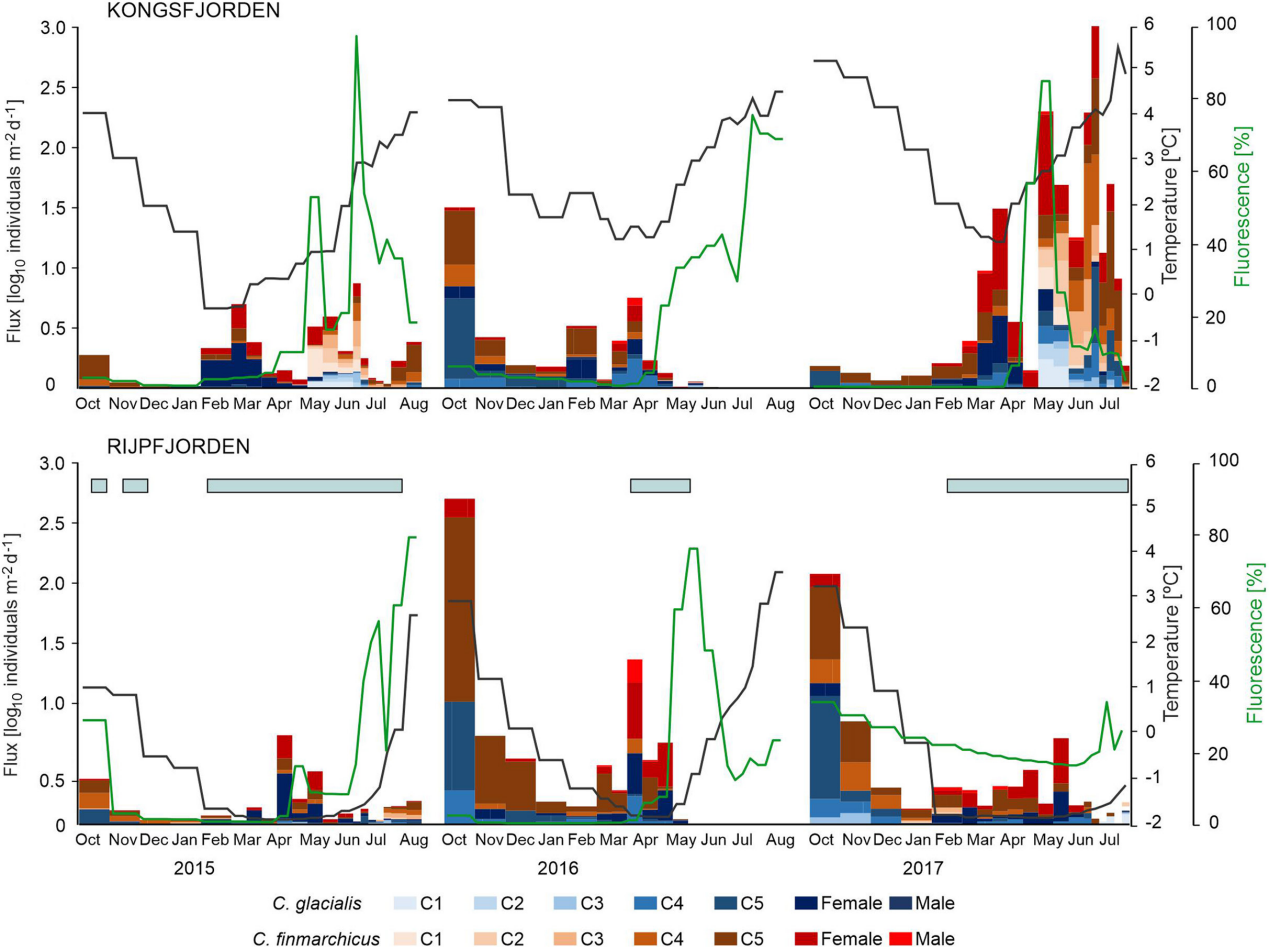
The flux of *Calanus finmarchicus* and *C. glacialis* development stages (copepodites C1–C5, females, and males), temperatures at a depth of 60 m (black line), and standardized fluorescence, averaged for the respective sediment traps exposition time, between October 2015 and August 2017. The presence of sea‐ice coverage in Rijpfjorden is shown as blue bars.

During the study period (October 2014 to August 2017), Kongsfjorden was largely influenced by water masses of Atlantic origin from October 2014 to August 2017. Mean temperatures <1°C were only observed during winter 2015 (Figure 2). Periods of relatively cold waters were shorter than in Rijpfjorden, with water temperature increasing from around April, and peaking in mid‐July. Summer 2017 was the warmest in the fjord, with mean temperatures >5°C in July and August. In Kongsfjorden, the peak of fluorescence was observed in early May and June in 2015, June and July 2016, and early May 2017 (Figure 2).

In contrast to the Atlantic‐influenced Kongsfjorden, the more Arctic Rijpfjorden was generally under the influence of cold (<1°C) water masses of Arctic origin, from autumn until sea ice break up in summer (Figure 2), with the duration of these periods differing between the sampling years. The first one was the longest and coldest, with mean temperatures between −0.9 and −1.8°C observed from November 2014 to the middle of July 2015 (Weydmann‐Zwolicka, Prątnicka, et al., 2021). During the warmest year, water temperatures stayed between −1.9 and 0°C between January and mid‐May 2016. The highest mean temperatures (over 1°C) were generally observed between August and October, as well as in late July and in November 2016. Based on upward looking ADCP measurements, Rijpfjorden was ice‐covered from mid‐October 2014 to late‐July 2015, from the beginning of April to the end of May 2016, and from mid‐February to mid‐July 2017. The peaks of fluorescence were linked with the presence of sea ice and observed in late June and August 2015, May 2016, and late June 2017.

### 3.2 Environmental factors and variables influencing *Calanus*

The daily flux of certain development stages of *Calanus* copepods differed between the fjords and seasons (Figure 2), although it is worth noticing that the population structure in both fjords was similar during each autumn, when the latest copepodites (C4–C5) dominated, and in winters, when the increasing proportion of adults was observed. Then, in the following seasons, the earlier copepodites (C1–C2) appeared in Kongsfjorden around May, while in Rijpfjorden, they were noted around July–August, after the breakup of the sea ice. Such a situation was observed in the two sampling years: October 2014 to August 2015 and October 2016 to August 2017. Interestingly, the proportions of *C. finmarchicus* and *C. glacialis* seemed to be similar in the respective periods in both fjords (Figure 2).

According to PERMANOVA, two tested single factors significantly affected the daily flux of *Calanus* development stages, e.g., calendar months, which explained 23.9% of variation in *C. finmarchicus* and *C. glacialis* development stages daily flux, and sampling years with nested months, responsible for 25% (Table 1). The fjord alone was not among the significant factors; however, through the interaction with the other factors, it significantly influenced the daily flux of the studied *Calanus* species.

**TABLE 1:** PERMANOVA table of results for the factors influencing the daily flux of *C. finmarchicus* and *C. glacialis* copepodites in Kongsfjorden and Rijpfjorden.

Factor	df	Sum of squares	Variability [%]	Pseudo‐*F*	Probability
Fjord	1	1626.1	0.6	0.86458	.548
**Month**	10	62,658	23.9	1.8654	**.004**
**Year (Month)**	19	65,670	25.0	2.3909	**.001**
**Fjord × Month**	10	35,806	13.7	1.7779	**.009**
**Fjord × Year (Month)**	18	35,711	13.6	1.3724	**.029**
Residuals	42	60,716	23.2		
Total	100	262,170	100.0		

*Note*: Significant predictors are given in bold.

**FIGURE 3:**
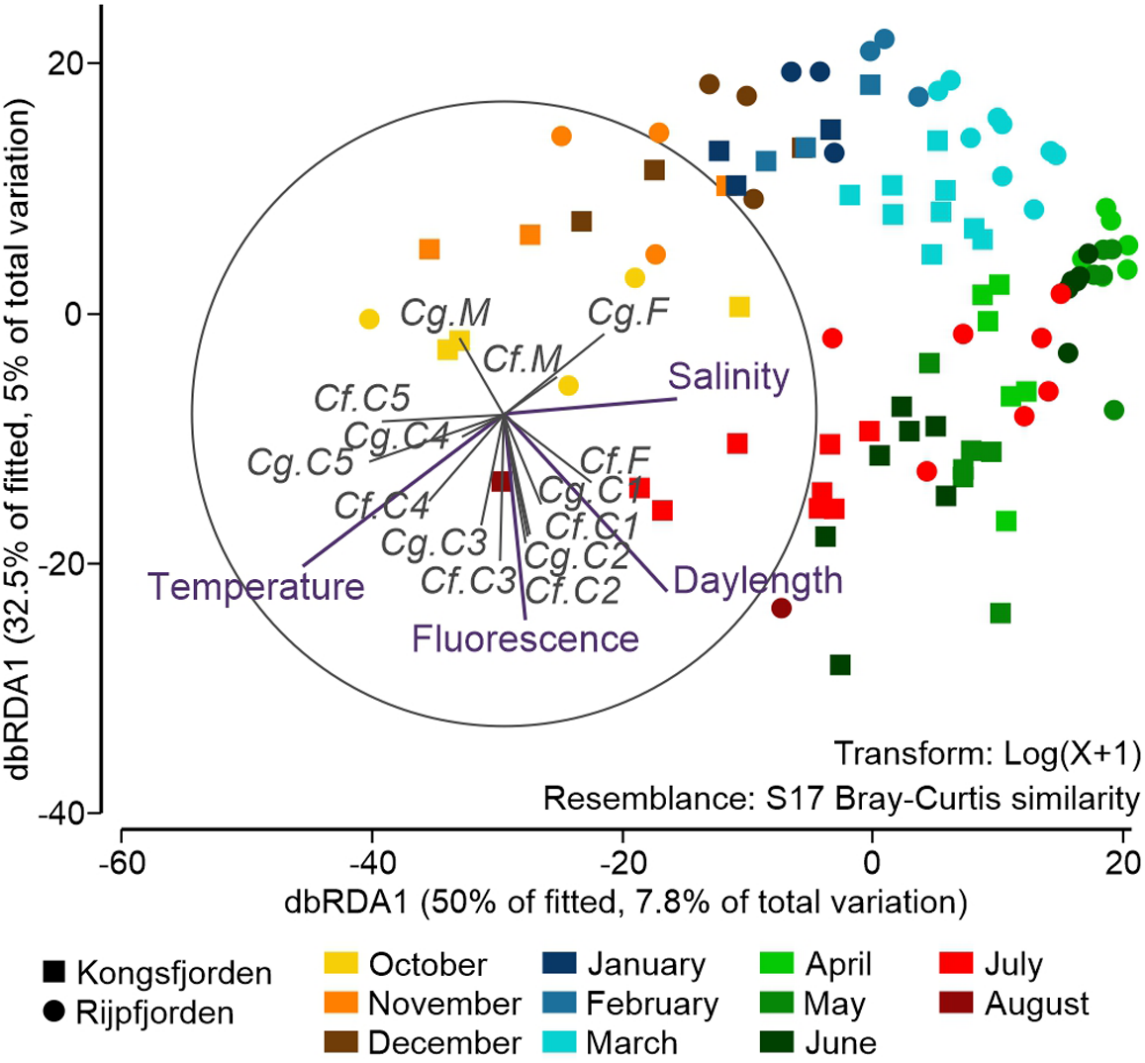
Distance‐based RDA plot illustrating the ordination of samples from Rijpfjorden (circles) and Kongsfjorden (squares), with environmental variables and *Calanus* (Cf, *C. finmarchicus* and Cg, *C. glacialis*) development stages (C1–C5; males, M; and females, F).

All tested continuous environmental variables significantly affected the daily flux of the studied *Calanus* development stages, although they explained reasonably low proportion of variance. The most influential variable was temperature, which was responsible for 5.39% variation in data, followed by daylength 5.09%, salinity 3.13%, and fluorescence 1.92% (Table 2). The importance of the first variable is well illustrated in the dbRDA ordination plot (Figure 3), in which the samples are distributed along the temperature gradient, with winter and spring samples from both fjords grouped opposite to the summer–autumn samples from Kongsfjorden, which were characterized by the highest temperatures. At the same time, the time shift between the fjords can be seen in the flux of *C. finmarchicus* and *C. glacialis* development stages, exemplified by the samples from June/July and November/December in Kongsfjorden located closely to the March and January/February ones from Rijpfjorden, respectively (Figure 3). Moreover, two main groupings of development stages can be observed in the ordination plot: earlier C1–C2 copepodites of both species with *C. finmarchicus* females in the spring–summer samples, collected in the period of permanent daylight and increasing fluorescence; and later, C4–C5 copepodites in the autumn, the period which was also characterized by the warmest water temperatures. On the other hand, the flux of *Calanus glacialis* females was negatively related to the temperature eigenvector, indicating their higher abundance in winter and early spring months.

**TABLE 2:** The results of DistLM sequential tests for the tested continuous environmental variables influencing the daily flux of *C. finmarchicus* and *C. glacialis* copepodites in Kongsfjorden and Rijpfjorden.

Variable	df	Adjusted *R* ^2^	Sum of squares	Variability [%]	Cumulative [%]	Pseudo‐*F*	Probability
**Temperature**	99	.044	14,118	5.39	5.39	5.635	**.001**
**Daylength**	98	.086	13,339	5.09	10.47	5.570	**.001**
**Salinity**	97	.109	8197	3.13	13.6	3.510	**.002**
**Fluorescence**	96	.120	5035.6	1.92	15.52	2.183	**.025**

*Note*: Significant predictors are given in bold.

### 3.3 Environmental niche overlap

**FIGURE 4:**
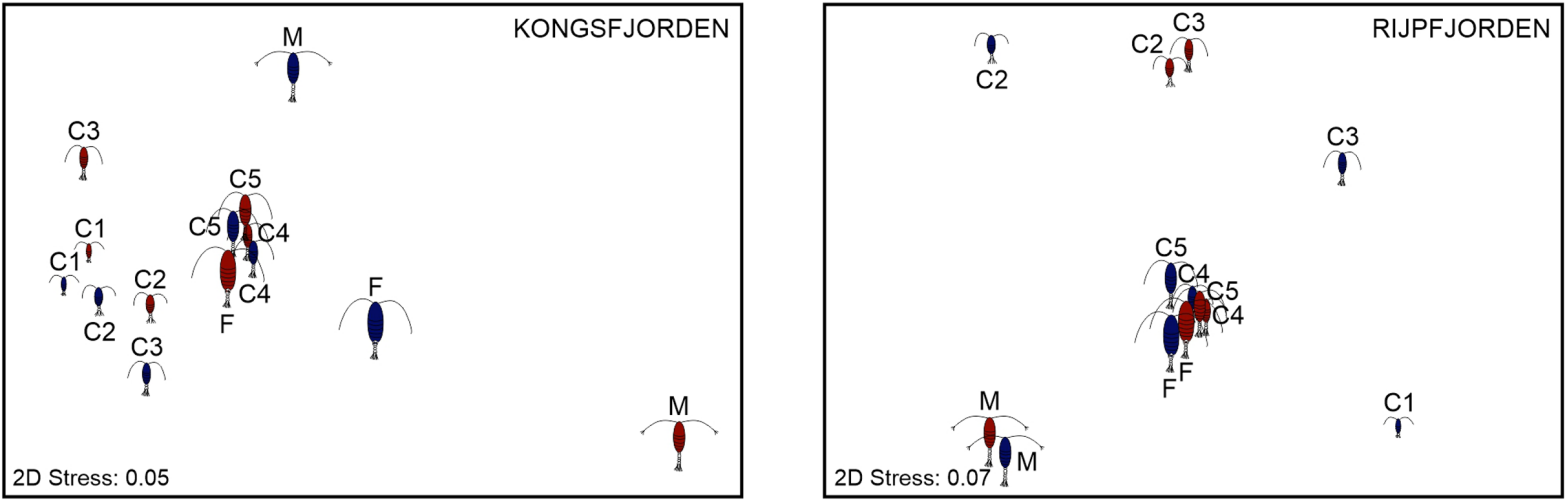
Similarities in ecological niche overlap between *Calanus finmarchicus* (red copepods) and *C. glacialis* (dark blue copepods) development stages (C1–C5; males, M; and females, F) in Svalbard fjords, represented graphically as nonmetric multidimensional scaling.

In Kongsfjorden, the environmental niches of *C. finmarchicus* males and earlier copepodite stages were significantly different and most dissimilar of all the developmental stages of both *Calanus* copepods (Table 3; Figure 4). According to the results for each axis/environmental variable separately, which are presented in the Supporting Information, the main differences between development stages in this Atlantic‐influenced fjord were also connected to salinity preferences, in which *C. finmarchicus* males differed significantly from the remaining copepodites of the same species (apart from C2), as well as from *C. glacialis* C4 and C5; while *C. glacialis* females differed from C1, C3, C4, C5 and females of *C. finmarchicus*, as well as from C4 and C5 of *C. glacialis* (Table S1a). Additionally, the males of the Atlantic species differed from its C3, C5 and females in terms of Chl‐a fluorescence preferences (Table S1c). Only two significant differences in temperature distributions were observed in Kongsfjorden: between *C. glacialis* C3 and its females and C5 of *C. finmarchicus* (Table S1b). Accordingly, in the nMDS plot (Figure 4), two main groups can be seen: (1) C4, C5, and females; and (2) C1–C3 of both species, with males being outliers. It is worth noting that fjords C4, C5 and females of the sibling *Calanus* copepods are clustered more closely than the other development stages, probably due to their high numbers in the autumn in both fjords.

**TABLE 3:** Mean niche overlap (upper) and its probability identified by the null model tests (lower) between the pairs of *Calanus finmarchicus* (Cf) and *C. glacialis* (Cg) development stages (copepodites, C1–C5; females, F; and males, M) in Kongsfjorden.

Stage	Cf C1	Cf C2	Cf C3	Cf C4	Cf C5	Cf F	Cf M	Cg C1	Cg C2	Cg C3	Cg C4	Cg C5	Cg F	Cg M
Cf C1		0.88	0.87	0.80	0.80	0.83	0.54	0.90	0.91	0.82	0.79	0.81	0.72	0.74
Cf C2	0.986		0.80	0.84	0.84	0.88	0.59	0.86	0.91	0.87	0.85	0.85	0.77	0.71
Cf C3	0.964	0.46		0.79	0.81	0.79	0.53	0.78	0.81	0.77	0.78	0.80	0.66	0.74
Cf C4	0.592	0.618	0.187		0.94	0.94	0.65	0.77	0.80	0.80	0.95	0.95	0.82	0.79
Cf C5	0.558	0.53	0.241	0.995		0.93	0.64	0.77	0.79	0.78	0.92	0.94	0.80	0.80
Cf F	0.763	0.897	0.177	0.996	0.987		0.65	0.80	0.83	0.81	0.93	0.93	0.84	0.76
Cf M	**0.045**	0.053	**0.022**	0.074	**0.045**	0.065		0.52	0.56	0.57	0.66	0.65	0.68	0.57
Cg C1	1	0.978	0.813	0.758	0.746	0.868	0.196		0.90	0.80	0.77	0.78	0.75	0.69
Cg C2	1	1	0.734	0.6	0.527	0.794	0.08	0.998		0.86	0.80	0.81	0.74	0.74
Cg C3	0.838	0.955	0.376	0.467	0.293	0.512	0.054	0.882	0.954		0.79	0.79	0.71	0.74
Cg C4	0.579	0.668	0.145	1	0.951	0.986	0.08	0.765	0.624	0.39		0.95	0.82	0.77
Cg C5	0.694	0.654	0.284	1	0.996	0.973	0.079	0.787	0.671	0.437	0.999		0.80	0.79
Cg F	0.214	0.209	**0.004**	0.154	0.081	0.295	0.118	0.673	0.306	0.108	0.225	0.096		0.68
Cg M	0.659	0.419	0.526	0.757	0.727	0.556	0.144	0.683	0.688	0.607	0.626	0.73	0.244	

*Note*: Significant differences are shown in bold.

Interestingly, none of the overall tested local realized niches between the six life stages of *C. finmarchicus* (C1 were not present in any sample) and seven of *C. glacialis* from Rijpfjorden differed significantly (Table 4). However, similarly to Kongsfjorden, the males of both species were most dissimilar with the niches of the earliest copepodites, C1–C2 (overlap ≤0.63, Table 3), while the local realized niches of C4, C5 and females of both species were most similar (overlap ≥0.88; Table 3), which is well illustrated in the nMDS plot (Figure 4), where they form a clear group. According to the analyses performed for each niche axis separately, the main dissimilarities in Rijpfjorden were connected to significant differences in salinity distribution between *C. finmarchicus* males and C3, C4, as well as *C. glacialis* C2, C3, and C5 (Table S2a). No significant differences between any development stage of any species were noted for temperature and fluorescence (Table S1b,c).

**TABLE 4:** Mean niche overlap (upper) and its probability identified by null model tests (lower) between the pairs of *Calanus finmarchicus* (Cf) and *C. glacialis* (Cg) development stages (copepodites, C1–C5; females, F; and males, M) in Rijpfjorden.

Stage	Cf C2	Cf C3	Cf C4	Cf C5	Cf F	Cf M	Cg C1	Cg C2	Cg C3	Cg C4	Cg C5	Cg F	Cg M
Cf C2		0.82	0.74	0.75	0.71	0.57	0.65	0.74	0.72	0.74	0.79	0.71	0.63
Cf C3	0.965		0.72	0.73	0.70	0.57	0.60	0.72	0.70	0.74	0.76	0.69	0.59
Cf C4	0.74	0.361		0.93	0.92	0.70	0.75	0.68	0.80	0.94	0.91	0.88	0.72
Cf C5	0.789	0.365	0.987		0.93	0.71	0.76	0.67	0.76	0.96	0.92	0.92	0.73
Cf F	0.644	0.273	0.953	0.976		0.73	0.73	0.66	0.76	0.92	0.88	0.93	0.73
Cf M	0.405	0.176	0.293	0.313	0.409		0.56	0.55	0.60	0.72	0.70	0.76	0.82
Cg C1	0.79	0.476	0.834	0.879	0.785	0.344		0.54	0.68	0.74	0.72	0.70	0.61
Cg C2	0.934	0.833	0.588	0.547	0.538	0.384	0.482		0.70	0.67	0.74	0.66	0.57
Cg C3	0.743	0.501	0.715	0.463	0.471	0.238	0.72	0.754		0.78	0.77	0.73	0.65
Cg C4	0.764	0.435	0.996	1	0.974	0.36	0.82	0.527	0.571		0.92	0.91	0.74
Cg C5	0.922	0.532	0.938	0.94	0.764	0.277	0.741	0.786	0.544	0.938		0.87	0.72
Cg F	0.622	0.233	0.701	0.92	0.967	0.555	0.658	0.535	0.324	0.9	0.611		0.76
Cg M	0.673	0.39	0.658	0.725	0.707	0.953	0.664	0.584	0.567	0.753	0.683	0.841	

Additionally, the RELATE routine performed on the matching resemblance matrices for *Calanus* niche overlap compared between Rijpfjorden and Kongsfjorden revealed high correlation in *C. finmarchicus* and *C. glacialis* development stages composition between the studied fjords (*r*_s_ = 0.53, *p* = .006).

**FIGURE 5:**
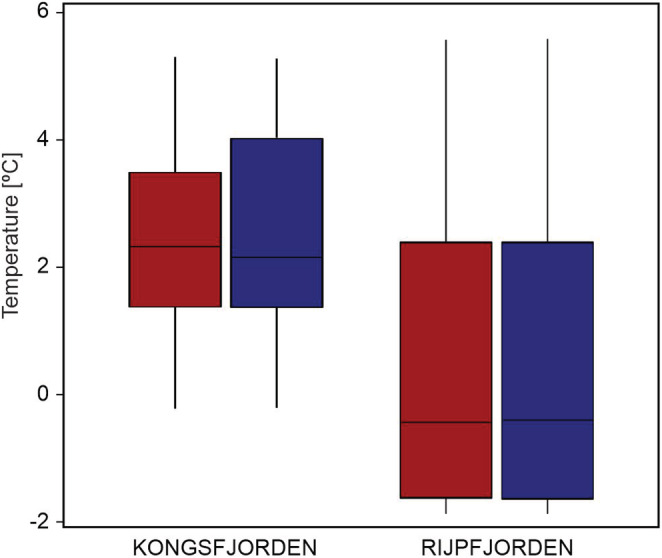
The average temperature niche overlap of *Calanus finmarchicus* (red) and *C. glacialis* (dark blue) in Svalbard fjords.

Consequently, according to the results of two‐way ANOVA, the only significant differences in terms of water temperature at 60 m, in the close proximity of sediment traps, were noted between the fjords, while there was no difference between the presence of the studied species in different temperature ranges. Interaction between Species × Fjord revealed that temperature distribution of the sibling *Calanus* species did not differ significantly within any fjord (Table 5, Figure 5).

**TABLE 5:** Two‐way permutated ANOVA table of results for temperature niche overlap of *Calanus finmarchicus* and *C. glacialis* in Kongsfjorden and Rijpfjorden.

Factor	df	Sum of squares	Variability [%]	*F* value	Probability
Species	1	2.8	0.7	0.857	.355
**Fjord**	1	577.9	56.9	179.292	**<.001**
Species × Fjord	1	0.3	0.1	0.094	.759
Residuals	524	1688.9	42.3		

*Note*: Significant predictors are given in bold.

## 4 DISCUSSION

In both investigated Svalbard fjords, the local realized environmental niches of *C. finmarchicus* and *C. glacialis* overlapped almost perfectly. Large similarities in population structure confirmed the synchronized development of *C. finmarchicus* and *C. glacialis* populations in high Arctic fjords, despite large differences in environmental conditions between the studied fjords: Arctic Rijpfjorden and Atlantic‐influenced Kongsfjorden. The main difference in the phenology of these sibling *Calanus* species between the fjords was linked to the presence of sea ice, which caused a 2‐ to 3‐month shift in age population structure, with an earlier start in the warmer and ice‐free Kongsfjorden compared to Rijpfjorden. A similar delay in zooplankton phenology between these fjords has been previously shown by Weydmann et al. (2013) and Weydmann‐Zwolicka, Prątnicka, et al. (2021), and was most likely connected to the onset of phytoplankton bloom, which was exploited by both species.

Although it is difficult to disentangle between *Calanus* species phenology and highly seasonal annual cycle in the high Arctic, what means that environmental conditions, and thus environmental niche, changes seasonally; it is striking that differences in temperature preferences of the sibling copepods were much higher between the studied fjords than between the species. Therefore, both sibling *Calanus* species exhibited similar phenology, and fulfilled their life cycles in Kongsfjorden and Rijpfjorden, despite contrasting environmental conditions in the fjords. No significant differences in temperature preferences between the co‐occurring Atlantic and Arctic species are in contradiction to most other zooplankton studies performed around Svalbard, in which higher abundance of *C. finmarchicus* was noted in the warmer fjord (Kongsfjorden), and/or during warmer periods in the colder Rijpfjorden, and the opposite in *C. glacialis* (e.g., Aarflot et al., 2018; Weydmann & Kwaśniewski, 2008; Willis et al., 2006). However, none of these studies quantified species niche overlap, and they were based on different data and statistical analyses, therefore likely methodological approach was responsible for the observed differences. The lack of environmental niche differentiation between the sibling *Calanus* species was also observed during year‐round study from Isfjorden, a fjord on the west coast of Spitsbergen, suggesting that both species may benefit from warming due to accelerated growth and higher survival of the recruits (Hatlebakk et al., 2022). The presented realized niches for *C. finmarchicus* and *C. glacialis* suggest high plasticity in both species. This is in agreement with Trudnowska et al. (2020) who reported high plasticity and synchronization in the population age structure between the two *Calanus* species in Hornsund fjord, and the adjacent southern part of the West Spitsbergen shelf.

Based on the similarities between the local realized niches, the main groups consisting of the latest copepodites (C4–C5 and females) of both *Calanus* species could be distinguished in both fjords in autumn–winter. Such large niche overlap, especially connected to temperature axis, and the correlation of the later copepodite stages to this variable revealed by dbRDA were connected with the fact that in autumn, when water temperatures in the fjords were the warmest, later copepodites were preparing to overwinter at greater depths (Falk‐Petersen et al., 2009). The distinguishable grouping of the earlier development stages in Kongsfjorden, observed mainly in spring, and the lack of such a grouping in Rijpfjorden were probably connected with food availability. Such variability in *C. glacialis* reproduction was reported by Daase et al. (2013), who noticed that in Rijpfjorden the Arctic copepod utilized the ice algae bloom to fuel spawning in spring. Conversely, in Kongsfjorden, *C. glacialis* was spawning earlier in the season, even in the absence of food, what allowed to support growth and development of the new generation by the phytoplankton bloom. Unfortunately, there were no reliable data on feeding conditions that could have been included to our analyses, although the measurements of chlorophyll *a* fluorescence that were taken during sediment traps exposure may still help to understand processes taking place in Svalbard fjords.

Importantly, it should be mentioned that the observed pattern of the prevailing flux of the later copepodites and the scarce presence of the earlier ones in the sediment traps installed at 60 m depth might be biased, due to distinct biology, mobility capabilities, and the noted depth preferences of *Calanus* development stages. Especially, data on the earliest copepodite stages, which usually feed on the blooms in the surface layers (Søreide et al., 2010), should be treated with caution, given the sediment traps' deployment depth and difficulties in distinguishing the earliest copepodite stages of *C. finmarchicus* and *C. glacialis* based on prosome length. Interpreting results only based on qualitative data at one specific depth, treated as a proxy of the position of the organisms in the water column, is one of the limitations of using sediment traps to collect zooplankton (Dezutter et al., 2019). However, the continuous collection of zooplankton and environmental data still seems to be a more complete method to quantify niche overlap between planktonic species than traditional single net tows. In fact, the use of automatic methods that allow for a continuous collection of zooplankton and environmental data is often the only available option in the high Arctic during late autumn–spring period. This is especially important in the era of recent rapid environmental changes, taking into account that most observations of zooplankton community in the Arctic are biased toward summer and autumn, which can skew the resulting data.

Given that *C. finmarchicus* and *C. glacialis* have largely overlapping niches in Svalbard fjords, what is the consequence for the ecosystem functioning? To assess that, we have to clarify what their individual role is for the ecosystem. The presence of large lipid‐rich individuals is one of the fundamentals of the energy transfer in Arctic marine ecosystems (Falk‐Petersen et al., 2009), and in that context, difference in size and energy content between the two species are relevant. The strategy of *C. glacialis* to grow slower but accumulate larger lipid reserves makes it an attractive, relatively large prey item for size selective predator (e.g., little auks) compare to its smaller and less lipid reach sibling species, *C. finmarchicus* (Karnovsky et al., 2003; Stempniewicz et al., 2021). And under the perception that energy content is solely species dependent, a change in the species composition can potentially have severe effects on energy transfer to higher trophic levels. With climate warming leading to less sea ice, increased water temperatures and longer algae growth season, there are indications that *C. finmarchicus* may become more successful in establishing itself in the Arctic, and a poleward shift and an increasing *C. finmarchicus* contribution to the overall *Calanus* biomass has already been observed in some Arctic regions (Aarflot et al., 2018; Chust et al., 2014; Hop, Wold, et al., 2019; Møller & Nielsen, 2020; Weydmann et al., 2014), thus changing prey size field and energy content for *Calanus* predators. However, so far there is no evidence that *C. glacialis* abundance or biomass is decreasing (Hop, Wold, et al., 2019; Møller & Nielsen, 2020). Furthermore, a trait‐based model suggested that climate‐driven changes in bloom phenology and temperature may drive the Arctic *C. glacialis* to adapt life history strategies similar to those of *C. finmarchicus*, i.e., shorter generation time and smaller body size (Renaud et al., 2018).

Thus, under climate warming scenarios, we can expect that the *Calanus* community in the Arctic will change toward populations consisting of individuals with smaller body size and less lipid content. But this may not be due to a change in species composition but due to changes in life history strategies in the co‐existing species. The large overlap in environmental niches between *C. finmarchicus* and *C. glacialis* observed in the present study suggests that both species may be able to adapt to highly variable environmental settings, not only on an interannual basis but also in a climate warming context, indicating some resilience in the *Calanus* community.

## AUTHOR CONTRIBUTIONS

**Finlo Cottier:** Conceptualization (equal); funding acquisition (equal); investigation (equal); writing – review and editing (equal). **Jørgen Berge:** Conceptualization (equal); funding acquisition (equal); supervision (equal); writing – review and editing (equal). **Sanna Majaneva:** Writing – review and editing (equal). **Piotr Kukliński:** Writing – review and editing (equal). **Adrian Zwolicki:** Formal analysis (equal); investigation (equal); writing – review and editing (equal). **Agata Weydmann‐Zwolicka:** Conceptualization (equal); formal analysis (equal); funding acquisition (equal); investigation (equal); project administration (equal); supervision (equal); visualization (equal); writing – original draft (lead).

## Supporting information


Appendix S1

## Data Availability

The detailed results of niche overlap analyses, as supplementary tables: Dryad https://doi.org/10.5061/dryad.1vhhmgqw6.
