# Supplementary material for: Environmental niche overlap in sibling planktonic species Calanus finmarchicus and C. glacialis in Arctic fjords
Source: Ecol Evol. 2022 Dec 8;12(12):e9569. doi: 10.1002/ece3.9569 (PMC9731911; doi:10.1002/ece3.9569)
Supplement: Supplementary file 1 — Appendix S1 [file ECE3-12-e9569-s001.docx]

**SUPPLEMENTARY TABLES**

**Table 1S.** Mean niche overlap (upper) and its probability identified by the null model tests (lower) between the pairs of *Calanus finmarchicus* (Cf) and *C. glacialis* (Cg) development stages (copepodites C1-C5, females F, and males M) in Kongsfjorden for the following niche axes:

**a) Salinity**

| Stage | Cf C1 | Cf C2 | Cf C3 | Cf C4 | Cf C5 | Cf F | Cf M | Cg C1 | Cg C2 | Cg C3 | Cg C4 | Cg C5 | Cg F | Cg M |
| --- | --- | --- | --- | --- | --- | --- | --- | --- | --- | --- | --- | --- | --- | --- |
| Cf C1 |  | 0.80 | 0.86 | 0.74 | 0.75 | 0.74 | 0.37 | 0.91 | 0.91 | 0.83 | 0.74 | 0.77 | 0.51 | 0.81 |
| Cf C2 | 0.74 |  | 0.76 | 0.90 | 0.91 | 0.92 | 0.51 | 0.83 | 0.84 | 0.89 | 0.90 | 0.90 | 0.66 | 0.75 |
| Cf C3 | 0.90 | 0.39 |  | 0.79 | 0.80 | 0.75 | 0.32 | 0.78 | 0.78 | 0.76 | 0.78 | 0.81 | 0.51 | 0.81 |
| Cf C4 | 0.44 | 0.94 | 0.33 |  | 0.96 | 0.95 | 0.49 | 0.77 | 0.78 | 0.85 | 0.92 | 0.94 | 0.70 | 0.77 |
| Cf C5 | 0.47 | 0.95 | 0.33 | 1.00 |  | 0.94 | 0.48 | 0.77 | 0.77 | 0.85 | 0.92 | 0.94 | 0.68 | 0.77 |
| Cf F | 0.42 | 0.97 | 0.20 | 0.98 | 0.96 |  | 0.53 | 0.77 | 0.78 | 0.87 | 0.92 | 0.92 | 0.72 | 0.74 |
| Cf M | **0.03** | 0.08 | **0.01** | **0.04** | **0.03** | **0.05** |  | 0.41 | 0.42 | 0.52 | 0.48 | 0.46 | 0.72 | 0.39 |
| Cg C1 | 0.99 | 0.90 | 0.76 | 0.71 | 0.68 | 0.70 | 0.16 |  | 0.97 | 0.88 | 0.76 | 0.78 | 0.54 | 0.77 |
| Cg C2 | 0.99 | 0.86 | 0.68 | 0.54 | 0.53 | 0.58 | 0.06 | 1.00 |  | 0.89 | 0.76 | 0.79 | 0.55 | 0.78 |
| Cg C3 | 0.86 | 0.95 | 0.50 | 0.78 | 0.77 | 0.88 | 0.12 | 0.97 | 0.97 |  | 0.82 | 0.83 | 0.66 | 0.81 |
| Cg C4 | 0.49 | 0.93 | 0.35 | 0.94 | 0.93 | 0.95 | **0.04** | 0.69 | 0.52 | 0.64 |  | 0.95 | 0.68 | 0.73 |
| Cg C5 | 0.54 | 0.94 | 0.43 | 0.98 | 0.98 | 0.92 | **0.02** | 0.73 | 0.61 | 0.70 | 0.99 |  | 0.66 | 0.76 |
| Cg F | **0.04** | 0.08 | **0.00** | **0.04** | **0.02** | **0.04** | 0.37 | 0.25 | 0.07 | 0.15 | **0.03** | **0.01** |  | 0.55 |
| Cg M | 0.86 | 0.64 | 0.80 | 0.64 | 0.62 | 0.50 | 0.08 | 0.84 | 0.79 | 0.85 | 0.50 | 0.59 | 0.15 |  |

**b) Temperature**

| Stage | Cf C1 | Cf C2 | Cf C3 | Cf C4 | Cf C5 | Cf F | Cf M | Cg C1 | Cg C2 | Cg C3 | Cg C4 | Cg C5 | Cg F | Cg M |
| --- | --- | --- | --- | --- | --- | --- | --- | --- | --- | --- | --- | --- | --- | --- |
| Cf C1 |  | 0.92 | 0.86 | 0.84 | 0.82 | 0.89 | 0.79 | 0.84 | 0.94 | 0.72 | 0.83 | 0.84 | 0.83 | 0.68 |
| Cf C2 | 0.98 |  | 0.80 | 0.77 | 0.74 | 0.83 | 0.75 | 0.81 | 0.95 | 0.77 | 0.77 | 0.76 | 0.79 | 0.61 |
| Cf C3 | 0.83 | 0.50 |  | 0.83 | 0.82 | 0.82 | 0.77 | 0.71 | 0.82 | 0.73 | 0.80 | 0.83 | 0.74 | 0.75 |
| Cf C4 | 0.73 | 0.22 | 0.44 |  | 0.93 | 0.94 | 0.86 | 0.73 | 0.80 | 0.67 | 0.95 | 0.94 | 0.82 | 0.78 |
| Cf C5 | 0.59 | 0.13 | 0.33 | 0.94 |  | 0.90 | 0.85 | 0.73 | 0.76 | 0.62 | 0.91 | 0.94 | 0.81 | 0.79 |
| Cf F | 0.91 | 0.47 | 0.36 | 0.93 | 0.65 |  | 0.84 | 0.79 | 0.84 | 0.67 | 0.93 | 0.91 | 0.87 | 0.72 |
| Cf M | 0.67 | 0.47 | 0.51 | 0.83 | 0.71 | 0.70 |  | 0.69 | 0.78 | 0.67 | 0.89 | 0.89 | 0.75 | 0.80 |
| Cg C1 | 0.89 | 0.80 | 0.50 | 0.50 | 0.52 | 0.67 | 0.52 |  | 0.83 | 0.59 | 0.74 | 0.73 | 0.88 | 0.55 |
| Cg C2 | 1.00 | 1.00 | 0.69 | 0.55 | 0.36 | 0.71 | 0.68 | 0.89 |  | 0.76 | 0.79 | 0.78 | 0.80 | 0.65 |
| Cg C3 | 0.41 | 0.46 | 0.30 | 0.10 | **0.04** | 0.09 | 0.30 | 0.31 | 0.54 |  | 0.66 | 0.66 | 0.59 | 0.62 |
| Cg C4 | 0.68 | 0.24 | 0.32 | 0.98 | 0.84 | 0.93 | 0.93 | 0.55 | 0.51 | 0.08 |  | 0.95 | 0.84 | 0.75 |
| Cg C5 | 0.71 | 0.20 | 0.49 | 0.95 | 0.97 | 0.83 | 0.94 | 0.52 | 0.49 | 0.09 | 0.98 |  | 0.80 | 0.80 |
| Cg F | 0.68 | 0.31 | 0.13 | 0.25 | 0.18 | 0.57 | 0.37 | 0.94 | 0.57 | **0.04** | 0.39 | 0.19 |  | 0.60 |
| Cg M | 0.44 | 0.24 | 0.50 | 0.58 | 0.62 | 0.37 | 0.81 | 0.34 | 0.39 | 0.26 | 0.48 | 0.61 | 0.16 |  |

**c) Chl-a fluorescence**

| Stage | Cf C1 | Cf C2 | Cf C3 | Cf C4 | Cf C5 | Cf F | Cf M | Cg C1 | Cg C2 | Cg C3 | Cg C4 | Cg C5 | Cg F | Cg M |
| --- | --- | --- | --- | --- | --- | --- | --- | --- | --- | --- | --- | --- | --- | --- |
| Cf C1 |  | 0.92 | 0.89 | 0.81 | 0.82 | 0.85 | 0.47 | 0.96 | 0.89 | 0.89 | 0.80 | 0.83 | 0.81 | 0.72 |
| Cf C2 | 0.91 |  | 0.84 | 0.86 | 0.86 | 0.89 | 0.52 | 0.93 | 0.94 | 0.95 | 0.87 | 0.88 | 0.87 | 0.76 |
| Cf C3 | 0.82 | 0.46 |  | 0.75 | 0.80 | 0.80 | 0.48 | 0.86 | 0.82 | 0.81 | 0.75 | 0.78 | 0.74 | 0.65 |
| Cf C4 | 0.41 | 0.51 | 0.09 |  | 0.92 | 0.93 | 0.60 | 0.83 | 0.84 | 0.88 | 0.97 | 0.98 | 0.94 | 0.83 |
| Cf C5 | 0.49 | 0.53 | 0.17 | 0.67 |  | 0.97 | 0.58 | 0.83 | 0.84 | 0.85 | 0.91 | 0.93 | 0.92 | 0.82 |
| Cf F | 0.62 | 0.70 | 0.19 | 0.78 | 0.97 |  | 0.57 | 0.85 | 0.87 | 0.89 | 0.92 | 0.94 | 0.94 | 0.83 |
| Cf M | 0.05 | 0.06 | **0.03** | 0.06 | **0.05** | **0.04** |  | 0.47 | 0.48 | 0.52 | 0.60 | 0.59 | 0.57 | 0.51 |
| Cg C1 | 1.00 | 0.99 | 0.80 | 0.70 | 0.71 | 0.84 | 0.17 |  | 0.91 | 0.92 | 0.82 | 0.84 | 0.83 | 0.75 |
| Cg C2 | 0.81 | 0.97 | 0.47 | 0.53 | 0.59 | 0.77 | 0.07 | 0.93 |  | 0.92 | 0.86 | 0.85 | 0.87 | 0.78 |
| Cg C3 | 0.82 | 0.99 | 0.39 | 0.72 | 0.58 | 0.79 | 0.07 | 0.96 | 0.93 |  | 0.89 | 0.89 | 0.88 | 0.78 |
| Cg C4 | 0.40 | 0.60 | 0.08 | 0.99 | 0.67 | 0.75 | 0.06 | 0.67 | 0.61 | 0.76 |  | 0.97 | 0.95 | 0.84 |
| Cg C5 | 0.51 | 0.61 | 0.13 | 1.00 | 0.82 | 0.89 | 0.06 | 0.74 | 0.63 | 0.75 | 0.98 |  | 0.93 | 0.82 |
| Cg F | 0.40 | 0.56 | 0.08 | 0.88 | 0.76 | 0.86 | 0.05 | 0.71 | 0.70 | 0.68 | 0.92 | 0.83 |  | 0.88 |
| Cg M | 0.41 | 0.44 | 0.20 | 0.64 | 0.61 | 0.62 | 0.13 | 0.58 | 0.59 | 0.52 | 0.67 | 0.62 | 0.86 |  |

**Table 2S.** Mean niche overlap (upper) and its probability identified by the null model tests (lower) between the pairs of *Calanus finmarchicus* (Cf) and *C. glacialis* (Cg) development stages (copepodites C1-C5, females F, and males M) in Rijpfjorden for the following niche axes:

**a) Salinity**

| Stage | Cf C2 | Cf C3 | Cf C4 | Cf C5 | Cf F | Cf M | Cg C1 | Cg C2 | Cg C3 | Cg C4 | Cg C5 | Cg F | Cg M |
| --- | --- | --- | --- | --- | --- | --- | --- | --- | --- | --- | --- | --- | --- |
| Cf C2 |  | 0.75 | 0.73 | 0.71 | 0.64 | 0.27 | 0.82 | 0.50 | 0.69 | 0.70 | 0.76 | 0.57 | 0.42 |
| Cf C3 | 0.84 |  | 0.64 | 0.64 | 0.55 | 0.22 | 0.60 | 0.47 | 0.50 | 0.66 | 0.67 | 0.51 | 0.31 |
| Cf C4 | 0.73 | 0.33 |  | 0.87 | 0.85 | 0.41 | 0.72 | 0.58 | 0.63 | 0.89 | 0.94 | 0.73 | 0.46 |
| Cf C5 | 0.65 | 0.30 | 0.77 |  | 0.90 | 0.50 | 0.75 | 0.50 | 0.54 | 0.96 | 0.91 | 0.82 | 0.56 |
| Cf F | 0.50 | 0.15 | 0.68 | 0.89 |  | 0.55 | 0.69 | 0.52 | 0.52 | 0.89 | 0.86 | 0.86 | 0.58 |
| Cf M | 0.08 | **0.01** | **0.03** | 0.08 | 0.16 |  | 0.36 | 0.16 | 0.17 | 0.49 | 0.42 | 0.65 | 0.61 |
| Cg C1 | 0.95 | 0.53 | 0.75 | 0.81 | 0.67 | 0.16 |  | 0.46 | 0.64 | 0.73 | 0.77 | 0.63 | 0.53 |
| Cg C2 | 0.51 | 0.39 | 0.50 | 0.32 | 0.37 | **0.04** | 0.46 |  | 0.62 | 0.51 | 0.56 | 0.44 | 0.20 |
| Cg C3 | 0.70 | 0.22 | 0.26 | 0.11 | 0.09 | **0.00** | 0.63 | 0.63 |  | 0.55 | 0.60 | 0.44 | 0.28 |
| Cg C4 | 0.66 | 0.37 | 0.87 | 1.00 | 0.87 | 0.07 | 0.78 | 0.34 | 0.12 |  | 0.92 | 0.80 | 0.53 |
| Cg C5 | 0.78 | 0.41 | 0.99 | 0.91 | 0.74 | **0.04** | 0.87 | 0.43 | 0.21 | 0.96 |  | 0.74 | 0.49 |
| Cg F | 0.34 | 0.10 | 0.18 | 0.39 | 0.73 | 0.29 | 0.46 | 0.23 | **0.03** | 0.40 | 0.22 |  | 0.66 |
| Cg M | 0.33 | 0.11 | 0.18 | 0.32 | 0.37 | 0.55 | 0.52 | 0.12 | 0.07 | 0.28 | 0.22 | 0.54 |  |

**b) Temperature**

| Stage | Cf C2 | Cf C3 | Cf C4 | Cf C5 | Cf F | Cf M | Cg C1 | Cg C2 | Cg C3 | Cg C4 | Cg C5 | Cg F | Cg M |
| --- | --- | --- | --- | --- | --- | --- | --- | --- | --- | --- | --- | --- | --- |
| Cf C2 |  | 0.87 | 0.59 | 0.63 | 0.57 | 0.67 | 0.42 | 0.88 | 0.58 | 0.63 | 0.69 | 0.62 | 0.65 |
| Cf C3 | 0.78 |  | 0.70 | 0.73 | 0.67 | 0.80 | 0.50 | 0.96 | 0.71 | 0.74 | 0.80 | 0.72 | 0.78 |
| Cf C4 | 0.29 | 0.26 |  | 0.96 | 0.96 | 0.85 | 0.73 | 0.71 | 0.89 | 0.96 | 0.90 | 0.97 | 0.83 |
| Cf C5 | 0.32 | 0.28 | 0.85 |  | 0.94 | 0.82 | 0.72 | 0.74 | 0.86 | 0.97 | 0.93 | 0.99 | 0.80 |
| Cf F | 0.28 | 0.24 | 0.86 | 0.67 |  | 0.82 | 0.74 | 0.68 | 0.87 | 0.93 | 0.87 | 0.95 | 0.81 |
| Cf M | 0.49 | 0.54 | 0.70 | 0.72 | 0.56 |  | 0.60 | 0.79 | 0.90 | 0.85 | 0.87 | 0.83 | 0.97 |
| Cg C1 | 0.28 | 0.30 | 0.52 | 0.49 | 0.58 | 0.46 |  | 0.52 | 0.65 | 0.71 | 0.67 | 0.73 | 0.58 |
| Cg C2 | 0.89 | 0.96 | 0.40 | 0.43 | 0.34 | 0.66 | 0.39 |  | 0.70 | 0.75 | 0.81 | 0.73 | 0.76 |
| Cg C3 | 0.41 | 0.39 | 0.94 | 0.87 | 0.81 | 0.82 | 0.54 | 0.47 |  | 0.88 | 0.86 | 0.87 | 0.90 |
| Cg C4 | 0.34 | 0.30 | 0.82 | 0.97 | 0.65 | 0.80 | 0.46 | 0.43 | 0.92 |  | 0.94 | 0.98 | 0.83 |
| Cg C5 | 0.43 | 0.43 | 0.49 | 0.62 | 0.32 | 0.88 | 0.38 | 0.57 | 0.68 | 0.68 |  | 0.92 | 0.84 |
| Cg F | 0.30 | 0.28 | 0.92 | 0.99 | 0.75 | 0.71 | 0.49 | 0.41 | 0.93 | 0.97 | 0.56 |  | 0.81 |
| Cg M | 0.55 | 0.61 | 0.91 | 0.95 | 0.78 | 1.00 | 0.56 | 0.72 | 0.86 | 0.97 | 0.98 | 0.94 |  |

**c) Chl-a fluorescence**

| Stage | Cf C2 | Cf C3 | Cf C4 | Cf C5 | Cf F | Cf M | Cg C1 | Cg C2 | Cg C3 | Cg C4 | Cg C5 | Cg F | Cg M |
| --- | --- | --- | --- | --- | --- | --- | --- | --- | --- | --- | --- | --- | --- |
| Cf C2 |  | 0.84 | 0.89 | 0.91 | 0.92 | 0.77 | 0.73 | 0.85 | 0.88 | 0.89 | 0.93 | 0.94 | 0.83 |
| Cf C3 | 0.84 |  | 0.82 | 0.82 | 0.86 | 0.69 | 0.70 | 0.74 | 0.89 | 0.83 | 0.80 | 0.84 | 0.68 |
| Cf C4 | 0.93 | 0.55 |  | 0.95 | 0.94 | 0.84 | 0.79 | 0.76 | 0.88 | 0.98 | 0.90 | 0.94 | 0.86 |
| Cf C5 | 0.97 | 0.57 | 0.97 |  | 0.94 | 0.81 | 0.80 | 0.78 | 0.88 | 0.94 | 0.91 | 0.95 | 0.83 |
| Cf F | 0.99 | 0.73 | 0.95 | 0.93 |  | 0.82 | 0.77 | 0.79 | 0.88 | 0.95 | 0.92 | 0.97 | 0.81 |
| Cf M | 0.72 | 0.37 | 0.72 | 0.57 | 0.57 |  | 0.71 | 0.72 | 0.73 | 0.83 | 0.81 | 0.81 | 0.88 |
| Cg C1 | 0.75 | 0.58 | 0.74 | 0.72 | 0.65 | 0.62 |  | 0.63 | 0.76 | 0.79 | 0.73 | 0.76 | 0.72 |
| Cg C2 | 0.95 | 0.69 | 0.68 | 0.71 | 0.74 | 0.62 | 0.59 |  | 0.76 | 0.76 | 0.86 | 0.81 | 0.76 |
| Cg C3 | 0.93 | 0.92 | 0.82 | 0.78 | 0.81 | 0.45 | 0.71 | 0.74 |  | 0.89 | 0.84 | 0.87 | 0.77 |
| Cg C4 | 0.94 | 0.58 | 1.00 | 0.94 | 0.96 | 0.66 | 0.70 | 0.66 | 0.86 |  | 0.89 | 0.94 | 0.85 |
| Cg C5 | 1.00 | 0.49 | 0.71 | 0.78 | 0.85 | 0.58 | 0.54 | 0.91 | 0.62 | 0.68 |  | 0.95 | 0.83 |
| Cg F | 1.00 | 0.64 | 0.93 | 0.96 | 1.00 | 0.57 | 0.60 | 0.78 | 0.78 | 0.94 | 0.95 |  | 0.82 |
| Cg M | 0.86 | 0.47 | 0.85 | 0.76 | 0.70 | 0.91 | 0.71 | 0.80 | 0.67 | 0.83 | 0.77 | 0.75 |  |
